# Thousands of RNA-cached copies of whole chromosomes are present in the ciliate *Oxytricha* during development

**DOI:** 10.1261/rna.058511.116

**Published:** 2017-08

**Authors:** Kelsi A. Lindblad, John R. Bracht, April E. Williams, Laura F. Landweber

**Affiliations:** 1 Lewis-Sigler Institute for Integrative Genomics, Princeton University, Princeton, New Jersey 08544, USA; 2 Department of Biology, American University, Washington, DC 20016, USA; 3 Department of Psychiatry, University of California, San Diego, California, La Jolla 92093, USA; 4 Department of Biochemistry and Molecular Biophysics; 5 Department of Biological Sciences, Columbia University, New York, New York 10032, USA

**Keywords:** *Oxytricha*, ciliate, long noncoding RNA, lncRNA, epigenetics

## Abstract

The ciliate *Oxytricha trifallax* maintains two genomes: a germline genome that is active only during sexual conjugation and a transcriptionally active, somatic genome that derives from the germline via extensive sequence reduction and rearrangement. Previously, we found that long noncoding (lnc) RNA “templates”—telomere-containing, RNA-cached copies of mature chromosomes—provide the information to program the rearrangement process. Here we used a modified RNA-seq approach to conduct the first genome-wide search for endogenous, telomere-to-telomere RNA transcripts. We find that during development, *Oxytricha* produces long noncoding RNA copies for over 10,000 of its 16,000 somatic chromosomes, consistent with a model in which *Oxytricha* transmits an RNA-cached copy of its somatic genome to the sexual progeny. Both the primary sequence and expression profile of a somatic chromosome influence the temporal distribution and abundance of individual template RNAs. This suggests that *Oxytricha* may undergo multiple rounds of DNA rearrangement during development. These observations implicate a complex set of thousands of long RNA molecules in the wiring and maintenance of a highly elaborate somatic genome architecture.

## INTRODUCTION

Long noncoding RNAs (lncRNAs), defined as transcripts >200 nt with no protein coding function, were once thought to represent primarily nonfunctional “junk” transcription. However, the discovery of ∼10,000 lncRNA loci in the human genome (Derrien et al. 2012) and evidence that lncRNAs play active roles in processes as diverse as chromatin remodeling (Gupta et al. 2010), transcriptional interference (Latos et al. 2012), and post-transcriptional modification (Yoon et al. 2012), suggests that they have important roles in biological systems that modern techniques are finally making amenable to study. Originally controversial, the idea of an RNA cache was proposed as a means of epigenetic transmission of sequence information across generations (Lolle et al. 2005). Our laboratory experimentally demonstrated that epigenetically inherited, maternal lncRNAs are essential for genome remodeling in the ciliate *Oxytricha trifallax* (Nowacki et al. 2008). Here, we present evidence that *Oxytricha* produces complete RNA copies of thousands of its somatic chromosomes during nuclear differentiation and development.

Like all ciliates, *Oxytricha* is a microbial eukaryote with two kinds of nuclei per cell. The smaller micronucleus (MIC) contains the germline, which provides haploid gametic nuclei for sexual conjugation. The larger macronucleus (MAC) contains the somatic genome, which is the source of gene transcription during asexual growth and reproduction. While the micronuclear genome consists of long diploid chromosomes, the MAC genome contains over 16,000 different chromosomes, most of which bear only a single gene and are a median length of 2515 bp (mean 3.2 kb) at a typical copy number of ∼1900n (Prescott 1994).

After sexual exchange, the exconjugant daughter cell produces a new MAC from a copy of the MIC through a series of dramatic genome rearrangements (for review, see Yerlici and Landweber 2014). This process eliminates 90%–95% of the MIC genome, including all satellite repeats, transposable elements, and germline-exclusive genes, as well as internally eliminated sequences (IESs) that interrupt the precursor gene segments in the MIC. These retained DNA regions are called the macronuclear-destined segments (MDSs). Short direct repeat sequences, called “pointers,” border consecutive MDS-IES junctions and may help guide the MDS joining events that build the somatic chromosomes. Approximately 20% of *Oxytricha*’s genes are “scrambled,” containing at least one MDS that is permuted or inverted in the MIC genome, relative to its location in the MAC (Chen et al. 2014). Because they have a different order or orientation in the MIC than in the MAC, they must rearrange before they recombine to form mature MAC chromosomes.

The process of genome remodeling in *Oxytricha* and other ciliates is guided by an RNA-based system of epigenetic inheritance. While the distantly related ciliates *Paramecium* and *Tetrahymena* use PIWI-associated “scnRNAs” to mark regions of the MIC for elimination (Mochizuki et al. 2002; Lepère et al. 2008) and other small RNAs that may facilitate IES removal (Sandoval et al. 2014; Noto et al. 2015), *Oxytricha* uses PIWI-associated RNAs (piRNAs) to mark segments of the MIC genome for retention rather than elimination (Fang et al. 2012; Zahler et al. 2012). These 27-nt piRNAs derive from both strands and show peak expression between 18 and 24 h after the beginning of conjugation (Fang et al. 2012; Zahler et al. 2012). Functional experiments demonstrated that injection of piRNAs targeting MIC-limited sequences can program retention of those sequences in the new MAC, and that the DNA sequence retention extends across sexual generations (Fang et al. 2012); however, piRNAs have not been demonstrated to program MDS joining or to be capable of substitution transfer to the rearranging molecule (Nowacki et al. 2008). Hence, the piRNAs appear to be incapable of programming DNA rearrangement (Fang et al. 2012).

**FIGURE 1.**
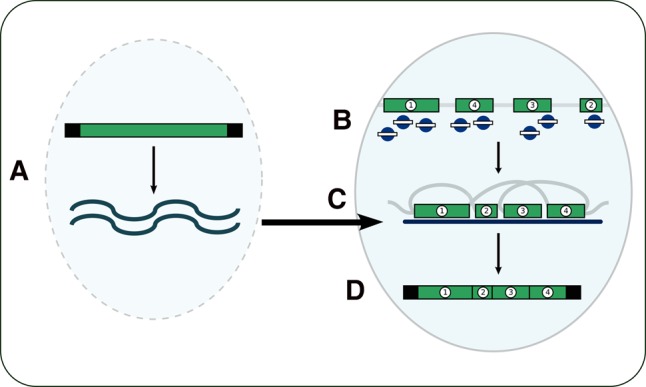
The template model of genome rearrangement. As the parental MAC degrades, (*A*) bidirectional transcription of the parental chromosomes produces lncRNA copies. In the developing MAC, (*B*) Otiwi1 (blue circles) associates with 27-nt piRNAs to mark MDSs (numbered) for retention. (*C*) The lncRNA templates are transported to the developing MAC, where they guide the correct configuration. (*D*) DNA breaks permit recombination between MDSs, with concomitant loss of MIC-limited DNA (light gray). Telomeres (black bars) cap sequence ends to form a mature MAC chromosome.

Long RNA copies of MAC chromosomes, on the other hand, can (re)program chromosomal rearrangements (Nowacki et al. 2008). *Oxytricha* produces these long RNAs during a burst of genome-wide bidirectional transcription early in cell development (Khurana et al. 2014); RT-PCR detects long, telomere-containing copies of whole chromosomes between 5 and 30 h after conjugation (Nowacki et al. 2008). Injecting synthetic RNA or DNA versions of a chromosome with incorrectly ordered MDSs leads conjugating cells to produce progeny whose chromosomes follow the aberrant ordering in their new MAC, and this effect persists across multiple sexual generations (Nowacki et al. 2008). Point mutations introduced by an injected RNA copy of a chromosome (Nowacki et al. 2008) and, in the related ciliate *Stylonychia lemnae*, substitutions in the telomeric sequence, can be passed to chromosomal telomeres and persist through multiple asexual generations (Fuhrmann et al. 2016). This suggests the long RNAs produced during conjugation may act as templates, as originally proposed in Prescott et al. (2003) and Angeleska et al. (2007), to guide rearrangement of genome segments during nuclear development (Fig. 1).

Here we report the first global survey of template RNAs in *Oxytricha trifallax* and the first such survey of telomere-to-telomere RNA transcripts in any eukaryote. We detect the presence of over 10,000 different lncRNAs corresponding to complete MAC chromosomes. These findings support the template RNA-guided model of DNA rearrangement and underscore the importance of long RNAs in the programming and maintenance of *Oxytricha*’s genome architecture.

## RESULTS

### *Oxytricha* produces thousands of full-length RNA copies of somatic chromosomes

**FIGURE 2.**
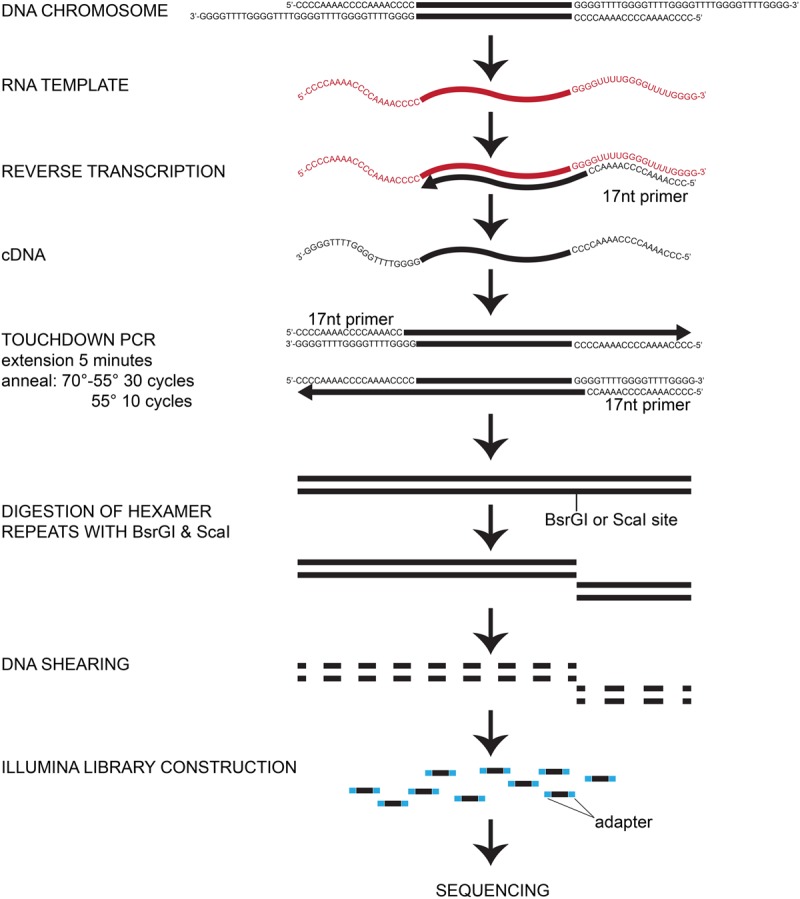
Strategy for genome-wide survey of template lncRNAs (Template-seq). Whole-chromosome RNA copies of somatic chromosomes were selectively amplified using telomeric primers and reverse transcribed into DNA. Contaminating hexamer repeat sequences were digested with frequent-cutting restriction enzymes, and the remaining sequences sheared and libraries prepared for Illumina sequencing.

**FIGURE 3.**
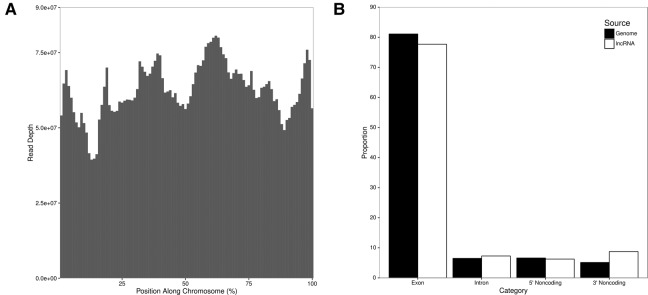
Template-seq reads cover entire chromosomes. While the aggregate of average read depth across all chromosomes (*A*) varies along the span of a chromosome, all portions are well covered, including noncoding, subtelomeric regions. There is no significant difference in the proportions of bases belonging to different sequence categories (*B*) between RNA-seq reads in this study and the genomic background, which indicates that template RNAs are noncoding, containing both introns and intergenic DNA, and that they can cover entire chromosomes from telomere to telomere.

**FIGURE 4.**
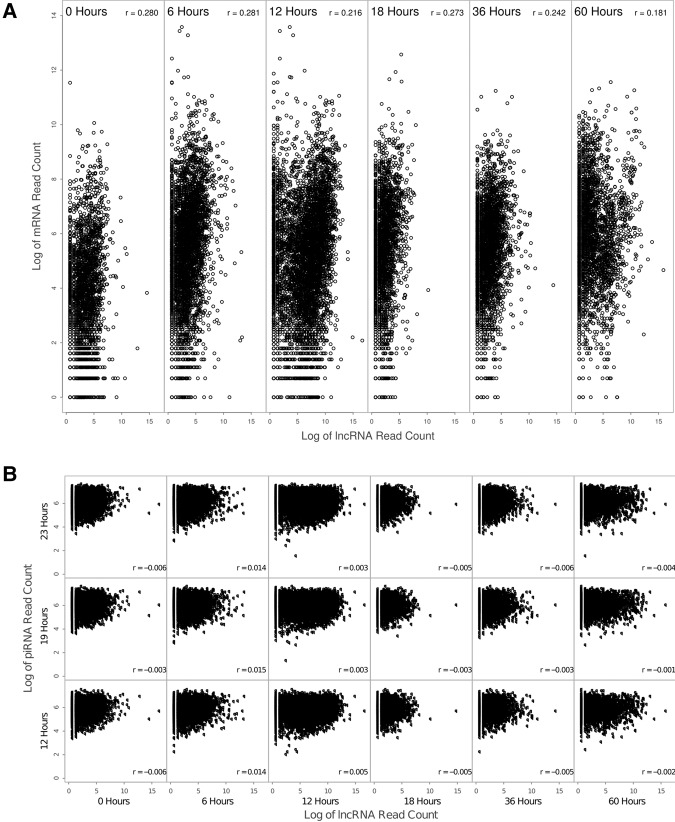
lncRNA levels show little correlation with the abundance of other RNAs. (*A*) The weak relationship between lncRNA levels and mRNA levels (Spearman's ρ = 0.181–0.281) across development indicates that our method captures noncoding RNA rather than mRNA and that template production is likely independent of normal gene transcription. (*B*) There is negligible correlation between lncRNA counts and piRNA counts at any point in development (Spearman's ρ = −0.009–0.088), which suggests that the two classes of RNA are largely independent, although it is possible that a subset of the longer RNAs are precursors to the piRNAs.

We used a novel PCR-based procedure (Fig. 2) to globally amplify RNA molecules containing telomeric repeats at both ends across a developmental time course. The six time points include: zero hours post-mixing (0 h), after compatible *Oxytricha* strains are combined but before mating begins; 6 h post-mixing, shortly after the first putative template RNAs were detected in Nowacki et al. (2008); 12 h post-mixing, which previous studies suggested might be the peak of template RNA production; 18 h post-mixing; and 36 and 60 h post-mixing, when few or no templates were previously observed. We sequenced the amplified RNAs using Illumina paired-end sequencing and mapped the resulting 100-bp reads to a subset of the *Oxytricha* MAC genome containing high-confidence chromosomes short enough to be amplified by the PCR step in the sequencing pipeline. Ultimately, we recovered RNA-seq read pairs corresponding to 10,507 different chromosomes, representing more than 2/3 of all completely assembled chromosomes in the somatic genome. This includes RNA-seq reads detected from 3230 chromosomes as early as the time of cell mixing (0 h), much earlier than expected. This set is enriched in chromosomes with a high level of expression during development, and transcription might occasionally begin at or within either telomere, where RNA polymerase localizes (Khurana et al. 2014), and extend to the other telomere, since subtelomeric regions are so short in *Oxytricha* (often <50 bp; Swart et al. 2013). Thus, in these cases, some reads could actually derive from mRNAs for genes with developmental expression. Overall, our RNA-seq data show little correlation with mRNA data collected over a similar developmental time course (Swart et al. 2013). In addition, our RNA-seq reads map across whole chromosomes, including introns and subtelomeric regions, with no enrichment for coding regions (Fig. 3), suggesting that mRNA contamination is not a major concern in our data set. Furthermore, fewer than 2% of reads map to germline-limited sequence at all time points. (The only exception is the presence in the 18 and 36 h time points of a germline-limited repeat that contains two telomere-like sequences and thus is captured by the sequencing pipeline.) This suggests that our experimental approach successfully captured noncoding RNAs that, as predicted, span entire chromosomes that contain telomeric sequences at both ends, and derive from the mature somatic genome rather than the germline. In addition, we found no correlation between lncRNA levels and piRNA levels at any time point in development (Fig. 4), suggesting that the molecules we recovered are a functionally distinct class of RNA, rather than merely piRNA precursors, but they could serve dual roles.

### The absence of some templates suggests that not all lncRNAs contain both telomeres

**FIGURE 5.**
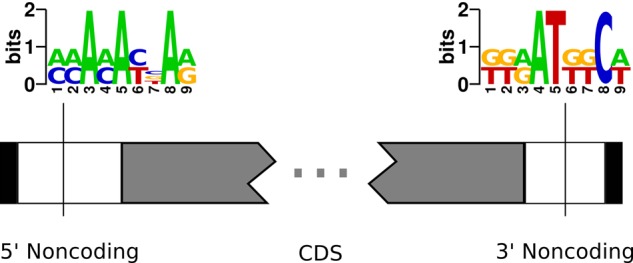
Motifs enriched in chromosomes without lncRNAs. Sequence logos indicate motifs significantly enriched in either the upstream or downstream noncoding regions of chromosomes for which our survey found no corresponding lncRNAs. Vertical bars indicate the median position of each motif within the respective noncoding region. If these motifs cause RNA polymerase to terminate transcription before reaching the end of a chromosome, it would result in templates that lack telomeres on both ends. Our RNA-seq pipeline would not detect such RNAs unless they contain an internal telomeric sequence.

To investigate why template RNAs were absent for a subset of *Oxytricha*’s MAC chromosomes, we searched for motifs associated with those chromosomes. This identified one motif enriched in the 5′ noncoding regions of nanochromosomes that had no identified templates, and a second motif enriched in the 3′ noncoding region of those same chromosomes (*P* < 2.2 × 10^−16^ for both motifs) (Fig. 5). Overall, nearly half (48%) of chromosomes with no mapped lncRNA reads had at least one instance of either motif, versus 37.0% of contigs with mapped lncRNA reads. Contigs without lncRNA data also have significantly more copies of the motifs per chromosome than those with lncRNA reads (one-sided Welch's *t* = 6.646, df = 3487.63, *P* = 1.738 × 10^−11^).

We propose that these motifs disrupt transcription of the template RNA and prevent RNA polymerase II (Khurana et al. 2014) from reaching the far telomere. The AT-rich 5′ motif, in particular, could mimic a transcription termination signal. Since, in principle, a template RNA only needs to span all of a chromosome's MDS junctions to guide DNA rearrangements, the presence of both telomeres may not be a strict requirement for function. However, because our survey only captured RNA molecules that contain telomeric sequences on both ends (see Materials and Methods), such prematurely terminated templates would not appear in our data set. Alternatively, either motif might interfere with the reverse transcription step in our protocol before PCR amplification, and this would also lead to their underrepresentation in the final data set; however, we find no significant difference in read number for contigs that contain at least one instance of either motif versus those without.

**FIGURE 6.**
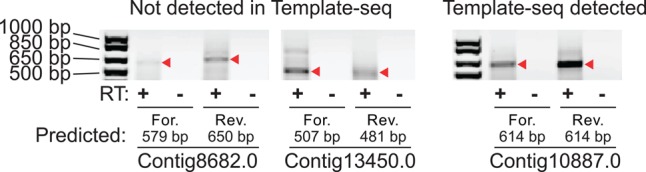
RT-PCR confirms the presence of template RNAs not detected in RNA-seq. On the *left* are two representative chromosomes that had no RNA-seq reads but were within the size range of our lncRNA sequencing method. On the *right* is a positive control amplification from a chromosome that did have RNA-seq reads. The combination of a gene-specific primer on one side plus a generic primer on the telomere side produces some background, as expected, but bands of the appropriate size are recovered in both forward (For.) and reverse (Rev.) reactions (marked with a red arrowhead). This suggests that the cell does produce template RNAs for these chromosomes, although it is possible that some only contain one telomeric sequence.

Furthermore, experiments using RT-PCR and gene-specific primers recovered sense, antisense, or both strands corresponding to lncRNA transcripts with a telomere sequence at one end for five (out of five) MAC chromosomes whose template RNAs were absent from our RNA-seq survey (two examples shown in Fig. 6). This evidence of RNA templates that our current methods were unable to detect is consistent with the proposal that the cell produces lncRNA transcripts for all of its chromosomes, and that these RNAs may include the presence of one or both telomeres.

### Heterogeneity in template abundance during nuclear development

**FIGURE 7.**
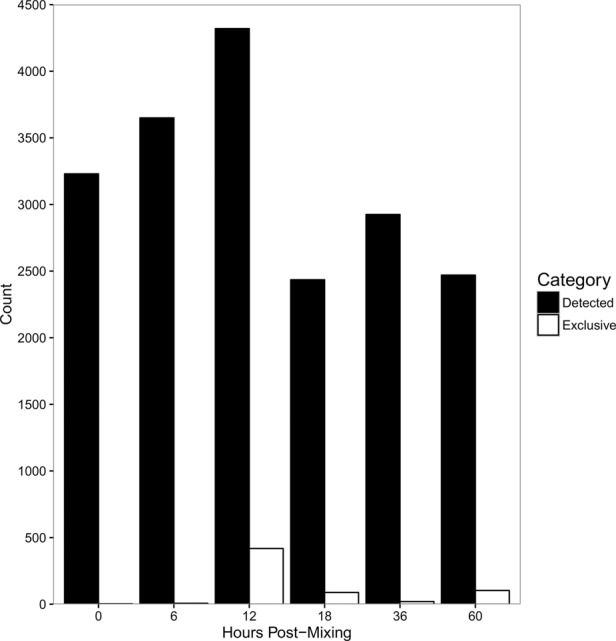
Different templates are present at different developmental stages. Thousands of template RNAs are present at every developmental time point, and each time point included a sub-population of template RNAs detected only at that time (white bars). Although the greatest diversity of templates is present 12 h post-mixing, hundreds of templates were detected only later in development.

**FIGURE 8.**
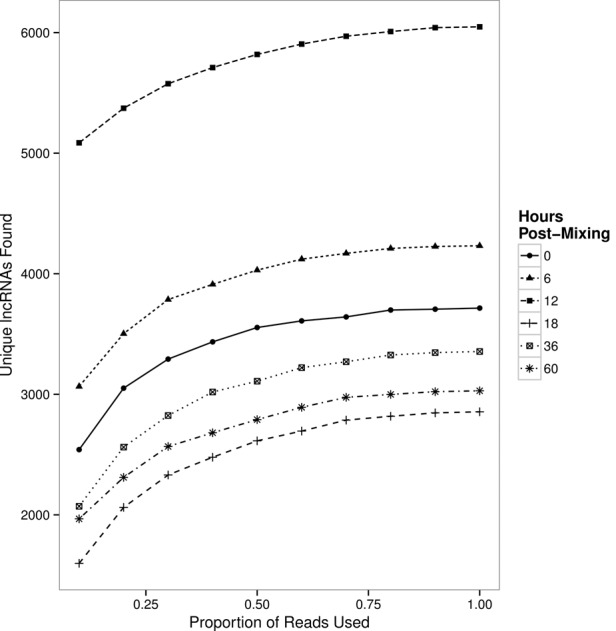
Subsampling analysis suggests that the lncRNA population is well-sampled. The number of chromosomes with corresponding lncRNA data saturates rapidly at all time points, with 60% of the data collected being sufficient to recover 90% of the unique lncRNAs at all time points. This suggests that additional read depth would not substantially increase the number of lncRNAs identified in this study, and that chromosomes with no lncRNAs identified in this study either are not transcribed into lncRNAs or were missed due to experimental limitations, rather than missed due to low abundance and stochastic loss.

We find that different populations of lncRNA templates with telomeric sequences at both ends are present at different developmental stages. While the greatest absolute number and diversity of templates appears 12 h post-cell mixing, thousands of templates appear only in samples from later time points. In addition, hundreds of templates were only detected at a single time point (Fig. 7), suggesting the possibility of rapid lncRNA production followed by swift degradation. While the telomere-to-telomere transcripts we surveyed are individually present at low abundance, resampling simulations indicate that our read depth was high enough such that we are not missing many lncRNAs as a result of stochastic loss (Fig. 8).

This variation in individual template RNA levels across development suggests that chromosome rearrangement may occur in waves, rather than all at once. Chromosomes whose templates appear in early development (at zero or 6 h post-mixing of mating types) have significantly higher RNA-seq expression levels during genome rearrangement (one-sided Welch's *t* = 3.450, df = 5181.362, *P* = 0.00028) than those whose templates were only detected later in development. Chromosomes whose gene products are required early might assemble first, especially if the cell relies on zygotic transcription from the developing macronucleus while the parental nucleus degrades. On the other hand, chromosomes that bear genes whose products are not required until vegetative growth, i.e., until nuclear differentiation is complete, are less critical and can be rearranged later during development.

In addition, some features of a chromosome's rearrangement map appear to correlate with when and how long its RNA templates are present. Chromosomes whose templates appear in only a single time point have a significantly greater number of pointers (the short direct repeats in the germline where somatic segments recombine) than those present at all time points (one-sided Welch's *t* = 2.183, df = 845.314, *P* = 0.01465), although they have fewer scrambled pointers, which require reordering during rearrangement, relative to chromosomes with templates found at all time points surveyed (one-sided Welch's *t* = −5.0907, df = 1842.702, *P* = 1.966 × 10^−7^). Those chromosomes whose templates are absent from our RNA-seq data set have a greater number of MDSs than those that are present (one-sided Welch's *t* = −33.718, df = 16281.52, *P* < 2.2 × 10^−16^) as well as more scrambled pointers (one-sided Welch's *t* = −3.645, df = 12449.62, *P* = 0.00013). Conversely, chromosomes with only a single MDS, which require no rearrangement or IES elimination but do require telomere addition, appear at significantly more time points than those with more than one MDS (one-sided Welch's *t* = 2.691, df = 10.086, *P* = 0.011). Overall, chromosomes that require the greatest number of MDS joining or rearrangement events have the most transiently available template RNAs.

## DISCUSSION

*Oxytricha* produces RNA copies of thousands of its somatic chromosomes during macronuclear development. The levels of these template RNAs fluctuate during development, and this temporal heterogeneity suggests that not all chromosomes undergo DNA rearrangement at the same time. Thus the corresponding need for template RNAs would vary for individual chromosomes.

Consistent with the findings of Khurana et al. (2014), both the absolute number of template RNAs and the number of distinct template RNA sequences peak 12 h after cell mixing, when RNA polymerase is found poised near both ends of the macronuclear chromosomes. However, we find some templates present earlier in the developmental cascade, even at the time of cell mixing. The early assembly of these chromosomes may be important for production of the corresponding gene products during development. Given that DNA replication is central to genome rearrangement in most ciliates (Ammermann et al. 1974), we hypothesize that the observed variations in genome rearrangement timing may reflect underlying variations in DNA replication timing, as reported in human (Koren et al., 2014).

If the cell produces template RNAs serially rather than through a single burst of transcription, then the time at which a chromosome's templates become available may influence the order of DNA deletion and descrambling events or template-guided DNA repair (Nowacki et al. 2008). Similarly, Möllenbeck et al. (2008) observed that chromosomes go through distinct stages of rearrangement, with simple DNA deletions often occurring before translocations during the process of DNA rearrangement. Furthermore, the data in Möllenbeck et al. (2008) are consistent with the possibility that some rearrangements might occur before RNA templates are abundant or even available, because the earlier DNA deletions were accompanied by higher levels of error at rearrangement junctions, which RNA template-guided DNA repair (Nowacki et al. 2008) may later restore. While there were no RNA studies in Möllenbeck et al. (2008), it suggested a temporal component to DNA processing, whereas the current study reveals a temporal component to RNA template presence.

In addition, we found that the persistence of template RNAs reflects the degree of fragmentation of the corresponding germline locus, and chromosomes that require a larger number of rearrangement events tend to have templates that appear for shorter durations. While one might expect chromosomes with more complex scrambling patterns to be more challenging (and time-consuming) to descramble and therefore require longer-lived templates, it is intriguing that such complex chromosomes are produced instead by some of the most transient lncRNAs in our data set. Overall, the heterogeneity of the lncRNA population during development suggests that different somatic chromosomes may differ in their rearrangement pathways, and that the entire population of molecules does not differentiate in lockstep. Such a strategy may reflect the cell's need to properly assemble over 16,000 chromosomes to build its somatic genome. In the future, it would be fruitful to extend the types of single-locus studies of DNA rearrangement pathways in Möllenbeck et al. (2008) to a genome-wide level of analysis, to test the hypothesis that the timing of chromosome rearrangement correlates with the timing of template RNA accumulation.

The relationship between template RNAs and the other RNA molecules that participate in genome rearrangement is also a subject of ongoing inquiry. On the basis of sequence, the telomere-to-telomere RNA transcripts of MAC chromosomes may perform myriad roles during post-zygotic development. For example, the lncRNAs not only guide DNA rearrangement, but also establish DNA dosage levels (Nowacki et al. 2010) and provide the possible precursors to piRNAs that protect regions of the germline DNA from elimination (Fang et al. 2012; Zahler et al. 2012). The present study identified template RNAs for both scrambled and nonscrambled loci, as well as for “IES-less” chromosomes that require no DNA deletion and only excision from the MIC genome plus telomere addition. Surprisingly, lncRNAs in the latter category appear to persist longer than those for chromosomes with multiple MDSs, though RNA templates in the related ciliate *Stylonychia lemnae* are also implicated in telomere sequence regulation (Fuhrmann et al. 2016).

While the ability of synthetic lncRNAs to reprogram MDS order (Nowacki et al. 2008) demonstrates their role in guiding DNA rearrangements, the observations that DNA rearrangements are highly error-prone during development (Möllenbeck et al. 2008) and that SNPs from injected template RNAs occasionally transfer to the MAC chromosomes of daughter cells (Nowacki et al. 2008) have implicated the lncRNAs in an additional role as templates for RNA-guided DNA repair (Nowacki et al. 2008). This step would allow the cell to identify and either repair or degrade aberrant rearrangements (Bracht et al. 2012) using the lncRNAs to proofread rearranging molecules (Möllenbeck et al. 2008). Therefore, late expressed templates might participate in one of the final steps of genome rearrangement, and the lncRNA templates surveyed in this study could both guide genome rearrangement and facilitate error correction.

## MATERIALS AND METHODS

### Cell culture and harvesting

*Oxytricha trifallax* mating compatible strains JRB310 and JRB510 were cultured in inorganic salt media according to established protocol (Chang et al. 2004) with *Chlamydomonas reinhardtii* and *Klebsiella oxytoca* as food sources. The cells were left overnight to exhaust their food, then filtered through gauze to remove any remaining algae before mixing them in equal proportions to initiate conjugation (Khurana et al. 2014). *Oxytricha* cells do not synchronize during conjugation. Cells were estimated to be within ∼6 h of one another in terms of development throughout the time course.

### RNA isolation and template amplification

**FIGURE 9.**
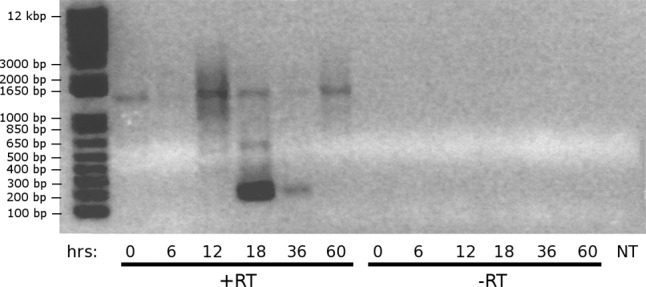
DNA contamination is low in lncRNA data. Lanes on the *left*, generated after reverse transcription with a telomeric primer, show amplification at all time points, whereas no product is visible in any of the lanes without reverse transcriptase. NT, no template negative control. Marker is 1 kb Plus DNA Ladder (ThermoFisher).

We collected whole-cell RNA from conjugating cells at six developmental time points (0, 6, 12, 18, 36, and 60 h post-mixing) using a TRIzol extraction kit (Invitrogen). We DNase-treated (Turbo DNAse) 10 µg RNA, precipitated it in phenol–chloroform, and resuspended it in 24 µL nuclease-free water (Agencourt Bioscience Corp., Beverly, MA). Reverse transcription reactions were performed with Superscript III enzyme on 3 µg RNA, using a telomeric primer (5′-CCCAAAACCCCAAAACC-3′). In addition, control reactions at each time point without reverse transcriptase showed no qualitative product (Fig. 9), but were sequenced as negative controls. For PCR we used eight replicates per time point to reduce jackpot effects. Fragments were amplified using FastStart enzyme with 0.5 µM of the same telomeric primer used for reverse transcription through 40 cycles of touchdown PCR (70°–55° for 30 cycles, then 10 cycles at 55°). Replicates were pooled and digested with BsrGI and ScaI-HF (NEB) to eliminate contaminating hexamer repeats observed in previous experiments (data not shown).

### Library preparation and high-throughput sequencing

Touchdown PCR products were sheared to 400 bp using the Covaris MiniTube system and gel purified with the QIAquick Gel Extraction Kit to obtain 350–450 bp fragments. Library preparation was carried out using a standard Illumina protocol, with end repair, A-tailing, and adaptor ligation, followed by a second round of gel purification. We eluted (Minelute, Qiagen) purified product into 10 µL nuclease-free water and amplified 5 µL by 12 cycles of PCR before gel purification, validation on a Bio-Analyzer, and sequencing on an Illumina GAIIx HiSeq instrument. Raw RNA-seq read files are available on SRA under accession SRP079066.

### RNA-seq analysis

We pruned the complete *Oxytricha trifallax* macronuclear genome (Swart et al. 2013) to a subset of 14,162 chromosomes considered high confidence (with both telomeres assembled) and less than 5000 bp in length (available at http://trifallax.princeton.edu/data/pacbio_twotelo_l5000bp.fa), as the incubation time we used for PCR precluded the capture of RNAs corresponding to longer chromosomes. Because template RNAs are expected to cover the entire chromosome without splicing, we used the nonspliced aligner BWA MEM (Li 2013) to align reads to the subset. Overall, we found 10,507 *Oxytricha* chromosomes with at least one mapped lncRNA read. From these we selected for further analysis (Figs. 3, 5, 7) a high-confidence subset of 4744 chromosomes with at least 16 pairs of reads mapped in the proper orientation and correct insert size and with a stringent threshold of at least 32-fold more mapped reads in the +RT data set as in the −RT data set.

### RT-PCR validation

We followed the protocol of Nowacki et al. (2008). Briefly, Turbo DNAse treatment (Thermo AM 2238) was followed by reverse transcription of 3 µg RNA isolated at 12 h using a long primer containing telomeric sequence and a “−RT” control. Subsequent to one-sided PCR, we used a short anchor primer plus one gene-specific primer to test forward and reverse strands independently. PCR was carried out with Phusion enzyme (NEB) for 35 cycles of: 98°C 10 sec (40 sec in the first cycle), 55° 30 sec, 72° 25 sec; followed by 72° for 5 min. Primer sequences are as follows (all 5′–3′):

Reverse transcriptase primer for anchor PCR
 ACTATAGGGCACGCGTGGTCGACGGCCCGGGCTGGTCCCCAAAACCCCAAAACCCCAAAA
Anchor primer
 ACTATAGGGCACGCGTGGT
Gene-specific primers:
Contig8682.0_Forward
 GGTTATTGATGCACTTAAATTACACTG
Contig8682.0_Rev
 CCACATGCATGATACTGGATTTTC
Contig13450.0_Forward
 CATATCAACGAGTTGAGAGAGATTC
Contig13450.0_Rev
 TCGAAGAAAGGCTTCTTGAATTGAG
Contig10887.0_Forward
 CTTAAGCTTTCCTGATTTAGTTCCTC
Contig10887.0_Rev
 CTCATAACTGCTCGACGGTTAAAC

### Motif finding

We limited our motif search to chromosomes with only one gene, as noncoding regions on multigene chromosomes are difficult to classify as either upstream or downstream: Some regions are 5′ to one gene while 3′ to another. The single-gene chromosomes were binned into two categories based on whether or not our survey found an RNA template for them at any time during development, the telomeres were removed, and the upstream (all sequence 5′ of the transcription start site) and downstream (all sequence 3′ of the transcription termination signal) regions were extracted. We also removed noncoding regions <5 nt long from the data set according to the requirements of the motif-finding algorithm. We used FIRE-1.1a (Elemento et al. 2007) for discriminatory motif finding with the parameters “--nodups=1” and “--exptype=discrete.”

### Statistical analysis

We used the R programming environment (R Development Core Team 2013) for all statistical analyses, with the ggplot2 package (Wickham 2009) for visualization and the subSeq package (Robinson and Storey 2014) for the resampling analysis. For all statistical analyses raw RNA read counts were normalized by library size, chromosome length, and DNA copy number.

### RNA data set comparisons

We used publicly available mRNA data from Swart et al. (2013) (downloaded from http://trifallax.princeton.edu/cms/databases/raw-data/transcriptome/reads/RNA-seq/WUGSC) and piRNA data from Fang et al. (2012) (GSE35018) for comparison with lncRNA levels during development.
